# A data-driven approach to prioritize MITRE ATT&CK techniques for active directory adversary emulation

**DOI:** 10.1038/s41598-025-12948-x

**Published:** 2025-07-30

**Authors:** Alshaimaa Abo-alian, Mahmoud Youssef, Nagwa L. Badr

**Affiliations:** Faculty of Computer and Information Sciences, Ain Shams University, Cairo, Egypt

**Keywords:** Adversary emulation, Active directory security, MITRE ATT&CK, Multi-Criteria Decision-Making (MCDM), Threat intelligence, Technique prioritization, Applied mathematics, Computer science, Information technology, Software, Statistics

## Abstract

Advanced Persistent Threats (APTs) continue to evolve, employing sophisticated and evasive techniques that pose significant challenges to modern defense mechanisms, particularly in Active Directory (AD) environments. Adversary emulation serves as a proactive security strategy, enabling organizations to replicate real-world adversary behaviors to assess and enhance detection, response, and mitigation capabilities. However, existing frameworks often lack a structured approach to prioritizing techniques based on impact, feasibility, and security control gaps, leading to suboptimal resource allocation. This study proposes a Multi-Criteria Decision-Making (MCDM) approach that integrates Operational Threat Intelligence (OTI) and structured datasets from MITRE ATT&CK to systematically prioritize adversary techniques. The methodology evaluates techniques across three key dimensions: Active Directory Impact, Threat Score, and Security Control Gap, employing entropy-based weighting to ensure an objective and data-driven prioritization process. To validate the proposed framework, a real-world case study based on the APT3 threat group is presented, demonstrating the applicability and effectiveness of the prioritization strategy in aligning adversary emulation with real-world attack scenarios. By focusing on high-impact and difficult-to-detect techniques, this framework enhances the effectiveness of adversary emulation and strengthens security postures in AD environments.

**Supplementary Information:**

The online version contains supplementary material available at 10.1038/s41598-025-12948-x.

## Introduction

A new line of security defenses is needed to combat todays cyberattack. The dynamic, evasive, resilient, and sophisticated nature of current threats is incompatible with traditional, static security approaches focused on heuristics and signatures^1^. To strengthen cybersecurity defenses and enhance blue team response to APTs, organizations should conduct adversary emulation exercises. Adversary emulation, a proactive approach, anticipates and simulates future adversary actions, addressing the limitations of traditional reactive methods that respond to past or ongoing attacks^2^. This method evaluates an organization’s security defenses by simulating actual cyberattacks, mimicking the tactics, techniques, and procedures (TTPs) of real or potential attackers, including virus attacks and phishing attempts^2^. This process identifies and remediates vulnerabilities before exploitation, thereby strengthening an organization’s security posture^2^. MITRE ATT&CK provides a comprehensive framework for analyzing the attack lifecycle and identifying platforms for adversarial behavior imitation^3^. While MITRE ATT&CK offers a structured framework for mapping adversary behaviors, its practical effectiveness significantly improves when combined with threat hunting, threat intelligence, and threat modeling^4^. In a proactive security approach, these elements serve unique yet complementary functions. Threat intelligence provides contextual information on adversaries, including their capabilities, motives, and frequently employed tactics, techniques, and procedures (TTPs), aiding decisions to prevent attacks or reduce detection time^5^. In contrast, threat hunting is a proactive cybersecurity strategy focused on mitigating, identifying, and searching for malicious threats that may have bypassed traditional security measures within an organization’s environment^6^. Similarly, threat modeling is a proactive, design-phase activity that analyzes potential threats across system contexts to assess risk and determine mitigations, typically during requirements or design stages, relying on a solid understanding of system architecture and attacker behavior^7^. Together, these practices inform and refine adversary emulation scenarios, ensuring they are grounded in realistic threats, aligned with environmental risks, and capable of revealing critical security gaps.

Operational Threat Intelligence (OTI) is most useful in adversary emulation, facilitating a deeper understanding of the “who,” “why,” and “how” of cyberattacks. This intelligence emphasizes attribution (“who”), motivations (“why”), and employed TTPs (“how”)^8^, offering contextual insights that help security teams understand adversary methodologies for strategizing and perpetuating campaigns. It incorporates insights into threat actor TTPs, enabling proactive defense against potential cyberattacks. The primary objective of OTI is to understand how threat actors are likely to attack an organization and align this understanding with effective mitigation and detection strategies^5^. Adversary emulation and mimicking adversary behaviors are crucial but can be resource-intensive and time-consuming if conducted randomly (scenario-based) or by following a specific emulation plan (e.g., APT29). As a purple team exercise, adversary emulation should be conducted properly to avoid these drawbacks. Consequently, the emulation process should be tailored to the specific environmental setup and existing security controls. Prioritizing emulated techniques based on recent research and frequently employed attack methods further increases the probability of successful operations. This approach also avoids mimicking worthless or legacy techniques; for instance, attempting to exploit a CVE associated with unpatched software in a fully patched system would be futile, as would trying to exploit LLMNR/NBT-NS Poisoning and SMB Relay in an SMB signed service, where the attack would fail due to signing, rendering emulation unnecessary. Despite existing emulation frameworks^2,9–11^, a prioritization process is often lacking, especially in complex environments like Active Directory. Addressing this gap is essential for relevant and efficient emulation efforts.

This study aims to bridge the Operational Threat Intelligence (OTI) perspective by focusing on TTPs and narrowing the scope to Active Directory, thereby improving Adversary Emulation through analysis and prioritization of the operational perspective with MITRE ATT&CK phases. Furthermore, this study introduces a data-driven approach to improve adversary emulation by prioritizing attack techniques based on real-world threat intelligence, exploitability, and environmental relevance. Instead of relying on static or scenario-based approaches, this methodology dynamically adapts emulation exercises to reflect the most critical threats within an organization’s Active Directory infrastructure. The main contributions of this paper are as follows:

- Integrating Operational Threat Intelligence (OTI) with Adversary Emulation to enhance realism and effectiveness by incorporating real-time insights into adversary tactics, techniques, and procedures (TTPs).
- Applying the MITRE ATT&CK framework to systematically identify, classify, and prioritize attack vectors specifically targeting Active Directory and Windows environments.
- Introducing a structured approach to selecting and simulating APT techniques based on impact, exploitability, and mitigating security controls, thereby improving adversary emulation efficiency.
- Contextualizing for Active Directory by defining and applying prioritization criteria (Potential Impact, Threat Relevance, Control Efficacy Gap) specifically relevant to AD security challenges and attack vectors.
- Enhancing emulation effectiveness with a practical approach for security teams to focus efforts on the most critical and relevant TTPs, optimizing resource allocation and improving AD security posture assessment.

The paper is structured as follows: Section ‎2 provides a comprehensive review of related work, covering adversary emulation concepts, AD security threats, the role of threat intelligence, and the application of MCDM in cybersecurity. Section 3 details the proposed MCDM-based prioritization methodology, including data acquisition and mathematical models. Section 4 presents an evaluation and case study demonstrating the application of the framework using APT3 data. Section 5 discusses the results, implications, limitations, and contributions of the proposed approach. Finally, Sect. 6 concludes the paper and outlines directions for future research.

## Related work

This section provides a review of the academic and industry literature pertinent to the core components of this research: adversary emulation, Active Directory (AD) security, the role of threat intelligence, and the application of Multi-Criteria Decision-Making (MCDM) in cybersecurity. The aim is to contextualize the proposed framework and identify the specific research gap it addresses.

### Adversary emulation: concepts, goals, and frameworks

Adversary emulation has emerged as a critical practice for proactively assessing and enhancing cybersecurity defenses against sophisticated threats like Advanced Persistent Threats (APTs)^12^. It involves simulating the Tactics, Techniques, and Procedures (TTPs) of real-world attackers to test detection capabilities, validate security controls, and train defenders^2,10,13^. The literature presents diverse approaches to conceptualizing and implementing adversary emulation, ranging from abstract simulations to automated execution frameworks and specialized documentation methodologies.

One stream of research focuses on simulating attacker behavior and network interactions. Drašar et al.^11^ introduced a session-level, event-driven simulator focused on analyzing attacker strategies and efforts based on varying entry points and credentials. This approach abstracts network interactions to the request-response level, offering insights into attacker progression but providing limited fidelity regarding specific operating system interactions or TTPs, particularly within complex environments like Active Directory (AD). While valuable for strategic analysis, its abstraction level distances it from the granular technique-level focus required for specific defense validation against known TTPs, such as those defined in the MITRE ATT&CK framework.

Moving towards more concrete TTP execution, several frameworks leverage the MITRE ATT&CK knowledge base, which has become a de facto standard for describing adversary behavior^2,10,14^. Karagiannis et al.^14^ proposed A-DEMO, a framework emphasizing the structured documentation of real-world cyberattacks (using a rootkit case study) by mapping attack phases directly to ATT&CK TTPs. The goal is to facilitate understanding, replication (emulation), and mitigation of documented attacks. While A-DEMO promotes standardized documentation and leverages ATT&CK effectively for describing known attack chains, it primarily focuses on replicating past incidents rather than proactively selecting or prioritizing techniques for specific environments or emerging threats. Its applicability to AD depends on the specific attack being documented.

Other approaches focus on automating the emulation process, often using ATT&CK as a library of executable actions. Applebaum et al.^10^ developed CALDERA, an influential automated adversary emulation system focused on post-compromise activities. CALDERA utilizes an automated planner built upon a logical encoding of the cyber environment and ATT&CK TTPs to intelligently select and execute actions to achieve predefined goals. This system is highly relevant to enterprise environments, often including AD, and its planning engine implicitly prioritizes actions based on goal achievement. Similarly, Ajmal et al.^2^ presented a threat-based adversary emulation approach, also leveraging ATT&CK, specifically for evaluating endpoint defenses in Windows environments. Their work emphasizes planning as part of the attack phase and includes algorithms for generating stealthy payloads, suggesting a prioritization based on effectiveness and evasion. Both CALDERA and the approach by Ajmal et al. represent significant steps towards automated, ATT&CK-driven emulation relevant to AD contexts. However, their prioritization mechanisms are either embedded within general planning logic^10^ or focused on payload generation and stealth^13^, rather than offering an explicit, configurable methodology for prioritizing techniques based specifically on the risks and characteristics of AD environments.

Addressing the challenge of realism and evasion, Orbinato et al. in^13^ introduced Laccolith, a hypervisor-based emulation solution designed for anti-detection. By injecting and executing malicious actions from below the guest operating system, Laccolith aims to bypass endpoint security controls (AV/EDR), thus providing a more realistic emulation of stealthy APTs. While capable of executing TTPs relevant to AD (e.g., credential dumping), its primary contribution lies in the evasion mechanism itself.

A distinct approach shifts the focus from individual TTPs or attack graphs to higher-level attacker objectives. Portase et al.^9^ proposed SpecRep, a system using a custom metalanguage derived from ATT&CK to define attack objectives. SpecRep compiles these objectives into multiple attack scenarios, exploring different combinations of actions (TTPs) to achieve the same goal. This objective-driven perspective inherently relates to prioritization, as achieving a specific objective (e.g., “Gain Domain Admin Access” in an AD context) requires selecting relevant TTPs. The system was demonstrated in heterogeneous infrastructures, including enterprise settings, but lacks a specific focus on AD intricacies or a dynamic prioritization mechanism beyond the initial objective definition.

In summary, existing research offers valuable frameworks and tools for adversary emulation, increasingly leveraging MITRE ATT&CK for structure and realism. Approaches range from high-level simulation^11^ and documentation^14^ to sophisticated automation^2,10^, objective-based planning^9^, and evasion techniques^13^. Several works^2,10^ are highly relevant to enterprise and AD environments and incorporate planning or threat-based selection, implicitly addressing technique prioritization. However, a specific gap exists in methodologies explicitly designed for prioritizing MITRE ATT&CK techniques based on the unique vulnerabilities, configurations, and high-value targets within Active Directory environments. Our work aims to address this gap by proposing a Data-Driven Approach for Prioritizing MITRE ATT&CK techniques leveraging Multi-Criteria Decision-Making (MCDM).

To clarify the distinctions and limitations across existing frameworks, Table 1 presents a comparative analysis of prior adversary emulation approaches, focusing on their primary objectives, integration of MITRE ATT&CK, applicability to Active Directory environments, and the presence or absence of explicit technique prioritization mechanisms.

**Table 1:** Comparative analysis of existing adversary emulation Frameworks.

Study	Methodological Focus	Integration of MITRE ATT&CK	Relevance to AD	Technique Prioritization	Emulation Fidelity
^11^	Network interaction simulation	None	Low	Not applicable	Abstracted (session-based)
^14^	Attack documentation/replication	Explicit mapping to TTPs	Conditional	Not applicable	Medium
^10^	Automated post-compromise emulation	Full ATT&CK TTP library	High	Implicit via goal planning	High
^2^	Endpoint defense evaluation under stealth constraints	ATT&CK-aligned TTP execution	High	Implicit by evasion logic	High
^13^	Evasion-focused low-level emulation	Direct TTP injection	Moderate	Not applicable	High
^9^	Objective-driven multi-path attack planning	Derived from ATT&CK objectives	Moderate	Implicit (goal-to-TTP compilation)	Medium

### Active directory security landscape

Recent CrowdStrike studies^15–18^ highlighted that adversaries are increasingly targeting identity-driven attacks, such as leaked account credentials, API keys, secrets, session cookies, tokens, one-time password (OTP) bypasses, and Kerberos-related vulnerabilities, including ticket manipulation. These attacks provide adversaries with the advantage of remaining stealthy and evading implemented defense mechanisms. Additionally, adversaries aim to minimize defenders’ network visibility by employing Living off the Land (LotL) binaries and therefore reducing potential indicators or alerts on the endpoint, which they recognize as being closely monitored. The studies also show that Kerberoasting attacks increased almost 600%. According to^15,19^ Active Directory (Domain Service) plays a crucial role for identity and access management in modern enterprises’ IT infrastructures, with 90% of Fortune 1000 companies relying on it. So, with the increasing number of identity-driven attacks and the reliance on Active Directory, once the adversary gains access to the system, they are poised to exploit this centralized infrastructure to escalate privileges, move laterally, and expand their control over the network. To increase persistence and bypass traditional detection methods, the attacker can use strategies such as Pass-the-Hash, or Silver Ticket. To gain control of sensitive corporate resources, they can also exert domain dominance by focusing on high-value accounts and group memberships. Since Active Directory is critical to an organization’s authentication and access control, compromising could have catastrophic effects that would allow APTs to achieve their goal.

For this reason, APT organizations target Active Directory to disrupt business operations, steal sensitive data or establish a persistent presence on an organization’s network. So, organizations should ensure that the Active Directory is adequately protected through strict monitoring, patch management and access controls to reduce the risk of attacks.

### Strategies for mitigating APTs in active directory environments

Most research on APTs has been conducted from a reactive approach, where response mechanisms have focused on when a breach has already happened. These studies emphasize the detection and mitigation of attacks after adversaries have gained access to AD, which is often a critical point in the chain of compromises for APT groups. This section discusses how recent research has adopted a reactive approach to mitigate APT attacks.

A. Binduf et al.^20^ explored the AD environment, its structure and components. Additionally, they analyzed a case study of a hypothesis Saudi company that does not implement AD as an identity access manager (IAM), identifying significant security risks due to the lack of centralized control over user access and resources. Consequently, they analyzed the most common active directory security issues, such as Service Accounts has over-permissioned, A lot of Domain Admins, Using Group Policy Preferences (GPP) to handling credentials, and the use of weak passwords for local administrator accounts. They also highlighted the reactive approach by emphasizing the alert feature, which is designed to enhance accountability by notifying administrators when critical changes or events occur within the AD environment. Therefore, they emphasized that the major issue in many companies is the improper configuration or underutilization of this alert feature, leading to gaps in monitoring and delayed responses to suspicious activities. Authors in^21^ also explored Active Directory environment and determined the characteristics, and the potential security risk in the environment. They also utilized an AI-methodological assistant to automate the process of security assessment. Furthermore, this framework was a combination of using machine learning and graph-based methods. The paper highlighted the importance of vulnerability assessment and penetration testing (VAPT) and demonstrating the effectiveness of using AI.

For credentials dumping phase in APT lifecycle, Mohamed et al.^22^ concluded that traditional signature-based detection methods often fail to detect APT attacks due to their stealthy and evolving nature. Therefore, they proposed the Strange Behavior Inspection (SBI) Model that focuses on behavioral anomalies during the credential dumping, by monitoring suspicious behaviors in RAM, CPU, Windows registry, and file systems. As a result, the SBI model reduces the detection time from 9 months to 2.7 min with a 99.8% accuracy rate, providing a reactive defense mechanism against APT attacks.

Collectively, these studies highlighted a reactive paradigm in which security measures are implemented after attackers have gained access to the network, especially the Active Directory (AD) infrastructure, which is frequently targeted in APT campaigns because of its centralized function in access control and authentication. Each work contributes to a growing body of literature that prioritizes post-compromise detection and response strategies, rather than preemptive adversary disruption. From static signature-based detection models to more dynamic behavior-driven systems, such research highlights both the strengths and limitations of reactive approaches.

To better contextualize the value of our proposed approach, Table 2 contrasts existing works based on key dimensions including defense strategy (reactive vs. proactive), relevance to Active Directory (AD), degree of automation, presence of a proposed tool or framework, and whether TTP prioritization is supported. A closer examination reveals that the majority of existing research adopts a reactive strategy, where detection and response mechanisms are triggered after adversaries have gained access to AD environments. Works such as^20,22–24^ emphasize monitoring, alerting, behavioral analysis, or signature-based detection, often relying on historical footprints of APTs rather than disrupting the attack lifecycle proactively. While^21^ employs AI to assist in security posture evaluation, its proactive aspects remain limited to posture auditing rather than adversary emulation or threat-driven defense planning.

In contrast, our approach represents a proactive shift, leveraging automation and a decision-making engine to prioritize TTPs specific to AD environments before an attack occurs. Unlike prior works, our method introduces a data-driven, Multi-Criteria Decision-Making (MCDM) mechanism to explicitly prioritize MITRE ATT&CK techniques based on AD-specific risks, configurations, and asset sensitivity. This not only facilitates targeted emulation but also allows defenders to focus on the most relevant and impactful techniques for validation or hardening. The table underscores a clear gap in proactive, TTP-prioritized frameworks tailored to AD, a gap our work aims to fill thus illustrates its unique contribution in both strategy and operational applicability.

**Table 2:** Comparative analysis of APT mitigation strategies in active directory Environments.

Paper	Defense Strategy	AD Focus	Automation	Tool/framework proposed	TTP prioritization
^23^	Reactive	**✓**			
^20^	Reactive	**✓**	**✓**		
^21^	Proactive	**✓**	**✓**		
^22^	Reactive		**✓**	**✓**	
^24^	Reactive			**✓**	
^25^	Reactive		**✓**	**✓**	
This work	Proactive	**✓**	**✓**	**✓**	**✓**

### Role of threat intelligence in proactive security

U.S. Department of Defense in^26^ demonstrates the relationship between data, information, and intelligence as a transformative process where raw data is processed into information, which is then analyzed to produce intelligence.

**Fig. 1:**
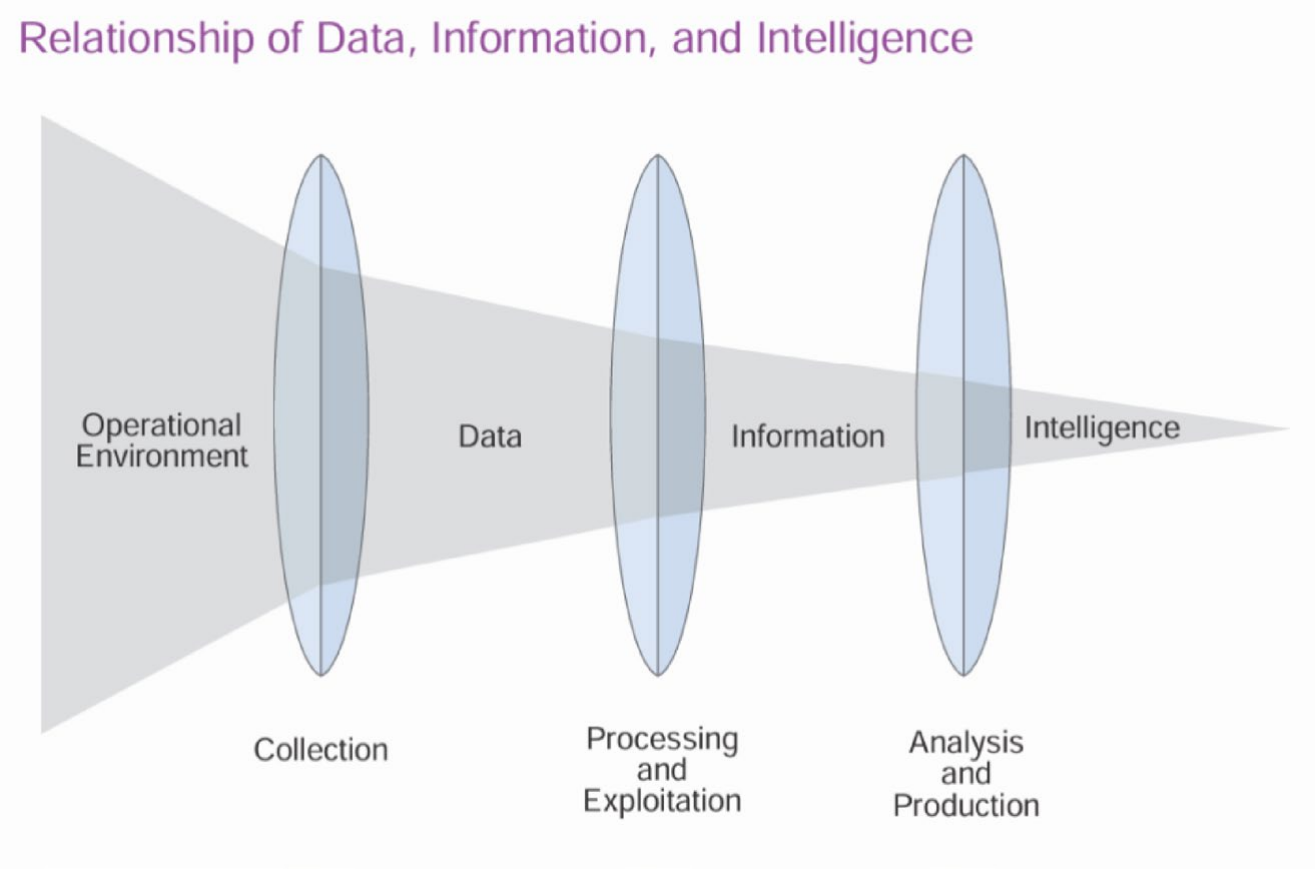
Relationship of Data, Information, and Intelligence.

This relationship is illustrated in Fig. 1, which highlights how data evolves into actionable intelligence through various stages^5^. Authors in^27^ also, describe this process of Threat Intelligence as the process of moving topics from *‘unknown unknowns’* to *‘known unknowns’* by discovering the existence of threats and then shifting *‘known unknowns’* to *‘known knowns’*, where the threat is well understood and mitigated.

Furthermore, Cyber Threat Intelligence (CTI) is a critical component of modern cybersecurity strategy, providing organizations with the knowledge and foresight needed to anticipate, detect, and respond to cyber threats. CTI is broadly categorized into three main types: Strategic, Operational, and Tactical, each serving a distinct purpose within an organization’s security posture. These types are differentiated by their scope, time horizon, audience, and technical depth, and together they form a hierarchical model of threat-informed decision-making.

#### Strategic threat intelligence

Strategic Threat intelligence is high-level, long-term insights into the larger cyber threat landscape are provided by strategic threat intelligence. Executive decision-makers, including risk management executives, chief information security officers (CISOs), and chief technology officers (CTOs), are usually informed by it. This type of CTI focuses on the geopolitical or economic variables that drive cyber threats, the reasons for adversaries’ targeting of particular industries, and how new trends like AI weaponization and cyberwarfare will gradually change the threat landscape^8,27^.

Typically non-technical, strategic CTI can be found in government advisories, intelligence briefings, or white papers. Although it is not immediately actionable at the technical level, it directs the development of policies, the distribution of resources, and risk management plans.

#### Tactical threat intelligence

Tactical threat intelligence has a short-term focus and is quite detailed. Through the use of certain Indicators of Compromise (IoCs), such as malicious IP addresses, domain names, URLs, file hashes, email subjects, or known malware signatures, it focuses on the prompt identification and mitigation of attacks^28^. For real-time blocking and alerting, this intelligence is frequently immediately fed into Security Orchestration, Automation, and Response (SOAR) platforms or Security Information and Event Management (SIEM) systems.

For frontline defenders like SOC analysts, IT security managers, and network defenders, tactical CTI is essential, but it is frequently very reactive and lacks context. Although it is quite effective at thwarting existing threats, it becomes less useful when faced with new or developing attack methods that circumvent static signatures.

#### Technical threat intelligence

Technical Threat Intelligence is occasionally categorized alongside tactical intelligence; however, it is more precisely concentrated on low-level, machine-readable data utilized in automated detection and the formulation of signatures. This domain encompasses YARA rules, Snort signatures, Sigma rules, exemplars of exploit code, malware sandbox evaluations, and decompiled code segments^5^. The principal beneficiaries of this intelligence are malware analysts, reverse engineers, detection engineers, and architects of SOC automation. It furnishes the detection logic that underpins alerts and the forensic insights requisite for comprehensive analysis. Moreover, it facilitates the development of tools, the creation of correlation rules, and the enhancement of tactical and operational intelligence outputs.

#### Operational threat intelligence

Operational Threat Intelligence occupies a position that interlinks strategic and tactical intelligence regarding its breadth and technical intricacy. It furnishes mid-term, contextually rich perspectives on the operational methodologies of adversaries, particularly their tactics, techniques, and procedures (TTPs), tools, campaign behaviors, infrastructural utilization, and exploited vulnerabilities. Operational Cyber Threat Intelligence (CTI) is predominantly utilized by Security Operations Centers (SOCs), Cyber Threat Intelligence analysts, and Incident Response Teams (IRTs)^8,27–29^.

The significance of operational CTI resides in its capacity to effectively bridge the divide between intelligence and actionable measures. It underpins detection engineering, red/purple teaming, threat modeling, adversary emulation, and the formulation of incident response playbooks. It augments an organization’s capability to foresee the behaviors of attackers, construct defenses around recognized intrusion sets, and evaluate security systems against adversary TTPs aligned with frameworks such as MITRE ATT&CK.

Operational Cyber Threat Intelligence (CTI) holds significant importance in the realms of threat detection and forecasting. Through the examination of the historical and current activities of threat actors, cybersecurity professionals are equipped to identify recurring patterns, deduce probable vectors of attack, and emulate adversarial tactics. For instance, an awareness that an Advanced Persistent Threat (APT) group frequently employs Mimikatz for the purpose of credential extraction, or exploits a specific vulnerability to facilitate lateral movement, empowers defenders to proactively monitor these activities within their own operational environments.

In order to enhance the understanding of the distinctions and practical implementations of the three fundamental categories of Cyber Threat Intelligence; strategic, operational, and tactical, a systematic comparison is delineated in Table 3. This evaluation examines each type of intelligence in accordance with essential criteria, which encompasses its primary emphasis, temporal scope, data classifications, application scenarios, technical intricacy, relevant stakeholders, and inherent limitations. The intention is to elucidate their respective functions within cybersecurity operations, underscore their alignment with various decision-making tiers within an organization, and accentuate the unique benefits of operational threat intelligence in facilitating threat detection, adversary simulation, and the engineering of detection mechanisms.

**Table 3:** Comparison of strategic, operational, and tactical threat Intelligence.

Criteria	Strategic TI	Operational TI	Tactical TI	Technical TI
Primary Focus	Long-term threat trends, geopolitical context, business risk	Adversary behaviors, TTPs, campaigns, tools	Immediate threat detection and response using IoCs	Low-level detection rules, malware code, exploit techniques
Time Horizon	Long-term (months to years)	Medium-term (weeks to months)	Short-term (real-time to days)	Very short-term (immediate to days)
Data Types	Industry threat trends, nation-state motivations, regulatory impacts	TTPs, malware families, adversary infrastructure, campaign patterns	IP addresses, URLs, file hashes, email subjects, malware signatures	YARA/Sigma rules, packet captures, malware sandbox results, code decomplication
Use Cases	Policy creation, strategic investment, risk management	Detection engineering, threat hunting, red/purple teaming, adversary emulation	SIEM correlation, firewall rules, IOC blocklists, incident triage	Malware analysis, rule writing, reverse engineering
Technical Depth	Low	Medium to High	High (but narrow)	Very High
Stakeholders	CISOs, CTOs, Board Members, Risk Managers	SOC teams, CTI analysts, Incident Responders, Detection Engineers	SOC analysts, IT security admins, network defenders	Malware analysts, reverse engineers, detection engineers
Actionability	Low (high-level insight)	High (maps directly to detection, mitigation, and testing)	Very High (real-time response)	Very High – Directly deployable in automated detection systems
Tools/Frameworks	Threat intelligence briefings, industry white papers	MITRE ATT&CK, CALDERA, MISP, YARA, OpenCTI	SIEMs, SOAR, AV signature databases, DNS blacklists	YARA, Snort, Zeek, Suricata, sandbox platforms (e.g., Cuckoo), IDA Pro, Ghidra
Limitations	Not actionable at the technical level	Requires expert analysis and contextual data for maximum value	Reactive and limited to known threats	Requires deep expertise, isolated in scope

### Multi-criteria decision-making (MCDM) in cybersecurity

Multi-Criteria Decision-Making (MCDM) provides a structured framework for evaluating and prioritizing options in complex decision environments, such as cybersecurity, where multiple conflicting criteria must be balanced. In cybersecurity, MCDM is increasingly applied to optimize resource allocation, prioritize threats, and enhance incident response. This section reviews the application of MCDM in cybersecurity, focusing on its use in security controls selection, alert prioritization, and threat prioritization, with an emphasis on the novel contribution of prioritizing MITRE ATT&CK techniques. MCDM in cybersecurity typically involves defining criteria (e.g., risk, cost, impact, likelihood), assigning weights to reflect their relative importance, and using a decision-making method to rank alternatives. Common MCDM methods include the Analytic Hierarchy Process (AHP), Technique for Order of Preference by Similarity to Ideal Solution (TOPSIS), and Weighted Sum Model. These methods enable decision-makers to systematically evaluate options, ensuring optimal use of limited resources.

Yevseyeva et al.^30^ applied MCDM to the selection of security controls under budget constraints, modeling the problem as a two-stage process. In the first stage, managers determine the security budget, treating loss prevention as a gain. In the second stage, the budget is allocated to controls using a portfolio optimization approach, balancing two criteria: expected return (gain from loss prevention) and risk (covariance of controls). The Sharpe ratio is used to select the optimal control portfolio from the Pareto front, solved via quadratic programming. This approach leverages financial portfolio theory, emphasizing control diversity to minimize risk, but does not address specific threat frameworks like MITRE ATT&CK.

Bassey et al. in^31^ utilized an MCDM-like approach for prioritizing security alerts in Security Information and Event Management (SIEM) systems. Their sliding window algorithm computes a priority score based on three criteria: the severity of the current alert, the criticality of affected entities, and the severity of previous alerts for the same entities. By averaging these factors, the algorithm ensures that alerts impacting critical systems are prioritized, even if their severity is lower. While effective for alert triage, this method lacks a formal MCDM framework and does not consider structured threat models like MITRE ATT&CK.

Almeida and Respício in^32^ proposed a decision support framework for selecting information security controls, leveraging ISO/IEC 27,001 and 27,002 standards to map vulnerabilities to mitigative controls. Their approach employs an integer programming model to optimize security portfolios by minimizing both implementation costs and expected losses from vulnerabilities. The framework was implemented as an Excel-based tool, enabling managers to evaluate controls and simulate scenarios. While effective for cost-benefit analysis and compliance alignment, the method relies heavily on predefined standards and does not incorporate dynamic threat intelligence or advanced multi-criteria decision-making (MCDM) techniques for evolving cyber threats.

In^33^ authors proposed a sector-based threat profiling approach for security control prioritization, leveraging MITRE ATT&CK and ETDA Threat Group Cards to map active threat actors’ TTPs to controls from ISO 27,001, NIST SP 800 − 53, and CIS frameworks. The methodology weights actors based on operation recency and newness, then prioritizes controls by their coverage of high-risk TTPs. While the approach automates threat-informed decision-making, it relies heavily on third-party data sources and lacks granularity in control effectiveness metrics. Unlike traditional vulnerability-centric models, this outside-in method aligns defenses with real-world adversary behaviors but may overlook organization-specific risks.

In contrast, the proposed research introduces a novel MCDM-based approach for prioritizing MITRE ATT&CK techniques, addressing a gap in structured threat prioritization. By selecting criteria such as threat impact, Prevalence, detection difficulty, and applying an MCDM method, the approach generates a ranked list of MITRE techniques tailored to an organization’s context. This enables targeted resource allocation and incident response, enhancing cybersecurity resilience. Unlike^30,33^, which focuses on control selection, or^31^, which addresses alert prioritization, this work directly tackles threat prioritization, leveraging the MITRE ATT&CK framework to provide actionable insights for defenders.

Our work proposes an MCDM-based approach to prioritize MITRE ATT&CK techniques for adversary emulation, using multiple criteria leveraging threat intelligence. It addresses inefficient, untargeted emulation by focusing on relevant threats, enhancing resource efficiency and tailoring defenses to organizational risks, thus strengthening resilience against advanced persistent threats (APTs).

The criteria adopted in this study significantly diverge from those employed in prior MCDM-based cybersecurity applications by emphasizing a threat-centric and adversary-informed perspective tailored to the context of adversary emulation. Previous works, such as^30,32^, largely focused on criteria related to static factors, implementation cost, compliance requirements, or control availability, framing security decision-making primarily around risk management or budget optimization. While effective in general control selection, such approaches often lack responsiveness to the dynamic and evolving nature of threat actor behavior.

In contrast, our study introduces a novel set of criteria specifically selected to enhance the fidelity and relevance of emulating real-world adversarial techniques, particularly in Active Directory environments. These criteria are aligned with both threat intelligence and operational feasibility, ensuring that the resulting prioritization supports proactive security testing and incident response preparation.

By framing criteria selection around both attacker intent and defensive capability, this approach improves upon prior MCDM applications by offering a dynamic, context-aware, and attack-surface-specific prioritization framework. This distinction is particularly critical for adversary emulation, where selecting techniques without considering attacker likelihood or detection challenges may lead to ineffective or unrealistic simulations. As such, the proposed criteria represent a substantial advancement over traditional MCDM configurations, aligning decision-making more closely with the realities of threat-informed defense.

In this study, entropy-based weighting is employed as the preferred method for determining the relative importance of decision criteria due to its objective, data-driven foundation and its alignment with the dynamic nature of threat intelligence-informed prioritization. In contrast to subjective weighting techniques, such as pairwise comparison matrices used in the Analytic Hierarchy Process (AHP) or expert-assigned static weights. Entropy-based weighting derives criterion importance directly from the inherent variability of the data. Specifically, it quantifies the degree of information dispersion (entropy) across alternatives, thereby assigning greater weight to criteria that provide higher discriminative power in the decision matrix.

This attribute is particularly advantageous in cybersecurity contexts, where the salience and influence of evaluation criteria can fluctuate based on evolving adversary behaviors, shifting threat landscapes, and organizational-specific vulnerabilities. By allowing the weighting to adapt automatically to data variability, the entropy method enhances the contextual relevance of the prioritization outcome without requiring manual tuning or subjective input.

Furthermore, the entropy approach contributes to methodological robustness by promoting scalability, reproducibility, and transparency, key requirements in empirical cybersecurity research. As the proposed approach is intended to support an automated and adaptable framework for the prioritization of MITRE ATT&CK techniques, entropy-based weighting ensures that the decision-making process remains unbiased, responsive to data changes, and grounded in quantitative analysis. Consequently, this methodological choice strengthens both the scientific validity and practical utility of the prioritization framework.

## The proposed data-driven prioritizing approach

**Fig. 2:**
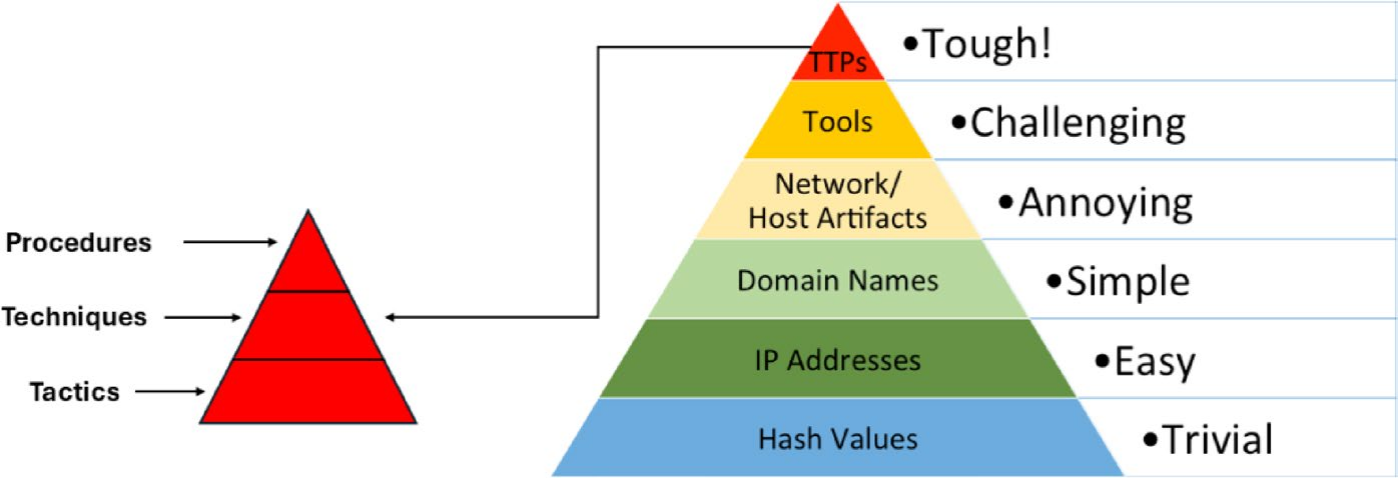
The detailed summit of Pyramid of Pain (TTPs Pyramid).

To emulate adversaries’ TTPs, we need to conduct a Cyber Threat Intelligence process first. This CTI process helps us understand which adversary actions are not only being observed in the wild but are also the most disruptive to the target organization. A fundamental concept in this context is the Pyramid of Pain introduced by David J. Bianco^34^, which illustrates how different types of Indicators of compromise (IoCs) vary in their impact on an adversary’s operations, as illustrated in Fig. 2. The detailed summit of Pyramid of Pain (TTPs Pyramid. At the base of the pyramid, items such as hash values, IP addresses, and domain names are relatively easy for attackers to change with little cost. Conversely, the top of the pyramid is occupied by adversary TTPs, such as specific attack techniques and procedures, that are more persistent and harder to alter and to detect. The proposed approach shifts the focus away from low-level IOCs toward the upper levels of the Pyramid of Pain, where TTPs reside.

The rationale for this focus is straightforward: while low-level indicators support detection, they do not capture the operational behaviors of adversaries. Accordingly, the approach emphasizes the top-level TTPs, refining them into a more structured hierarchy, as illustrated in Fig. 2 within this hierarchy:

- **Tactics** (e.g., “Credential Access”) represent broad objectives adversaries aim to achieve and are the easiest to replicate since they define “what” rather than “how.”
- **Techniques** (e.g., “Credential Dumping”) describe specific methods used to achieve a tactic, such as extracting credentials from memory or files, offering a moderate level of flexibility and challenge.
- **Procedures** are the most intricate, detailing the exact execution steps, tool configurations, and adaptation mechanisms required to operate within a given environment. For instance, dumping LSASS (Local Security Authority Subsystem Service) memory using tools like Mimikatz or ProcDump requires environmental knowledge, proper tool configurations, and operator expertise to bypass security controls.

**Fig. 3:**
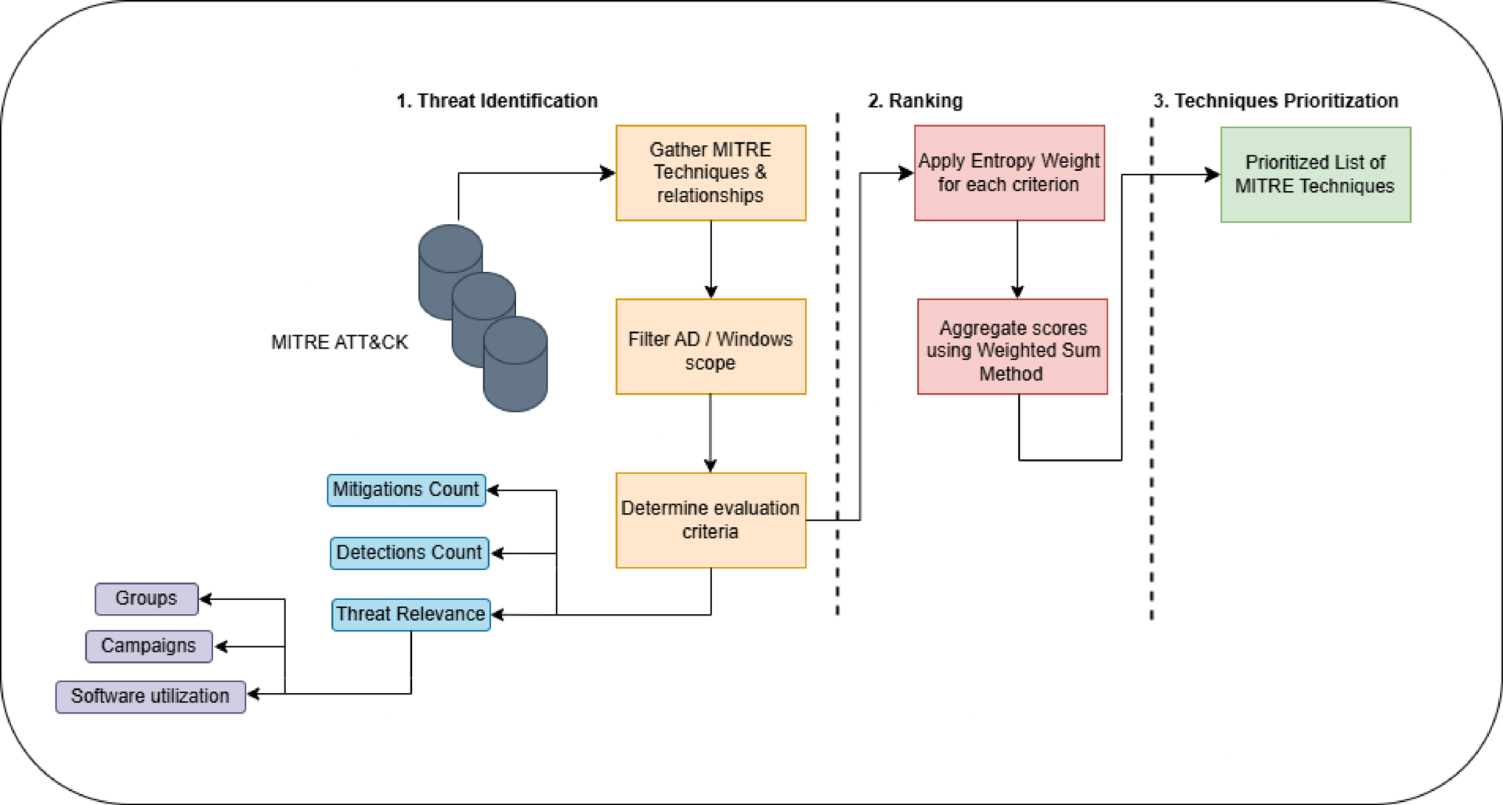
The Proposed Approach for Technique Prioritization.

Utilizing Operational Threat Intelligence (OTI) enhances this structured approach by providing real-time insights into adversary operations. By mapping TTPs effectively, OTI enables both strategic prioritization (understanding high-level adversary objectives) and tactical mitigation (identifying specific steps for detection and response). This process directly informs adversary emulation exercises, strengthening detection systems while continuously updating intelligence to refine security postures. The structured approach adopted in this work follows a systematic prioritization pipeline, as illustrated in Fig. 3. This figure outlines the key stages of the prioritization process, linking CTI insights with Multi-Criteria Decision-Making (MCDM), Entropy Weighting Method, and Weighted Sum method for ranking high-risk TTPs relevant to Active Directory environments. The framework consists of three primary phases:

- Threat Identification, in this phase, MITRE techniques and relationships are collected, then filtered out non-AD techniques, and finally, the evaluation criteria are calculated, which consist of the count of mitigations, detections, threat groups, campaigns, and the software utilizations.
- Subsequently, in the ranking phase, Entropy weighting method is used to weight the score of each technique based on the evaluation criteria. In addition to that, the weighted sum method is used as Multi-Criteria Decision-Analysis method.
- At the final stage, the techniques are ranked based on the priority score.

With a structured understanding of adversary tactics and their operational significance, the next phase focuses on the analysis and prioritization of TTPs leveraging MITRE ATT&CK. The proposed approach adopts MITRE ATT&CK as the foundational framework for threat research. To establish an effective prioritization process, an initial analysis of MITRE ATT&CK is conducted, focusing on TTPs relevant to Active Directory and Windows environments. This ensures that the prioritization model is aligned with the threats most pertinent to enterprise infrastructures, facilitating a more targeted and actionable adversary emulation strategy.

### Data acquisition and Preparation

The proposed approach utilizes MITRE ATT&CK to systematically analyze and prioritize adversary TTPs. Furthermore, to establish an effective prioritization process, an initial analysis of MITRE ATT&CK is required, focusing on TTPs relevant to Active Directory and Windows environment. In^35^, B. Al-Sada et al., analyzed the MITRE ATT&CK framework, covering domains, software, groups, campaigns, techniques, and tactics. However, the data set used in their study is not publicly available and can only be requested via email from the authors. To address this limitation, the proposed approach introduces a comprehensive analysis focusing on TTPs and Active Directory. Additionally, the dataset used in this research has been made publicly available, with detailed analysis steps.

Moreover, AttackCTI (Attack Threat Intelligence Collector Tool), version 0.5.4 – MITRE ATT&CK TAXII 2.1(OTRF, n.d.) is utilized to access up-to-date MITRE ATT&CK content in STIX format via a public TAXII server. AttackCTI is an open-source tool available at: https://github.com/OTRF/ATTACK-Python-Client.

### Methodology of MITRE ATT&CK analysis

To implement the proposed approach, the MITRE ATT&CK framework is analyzed using Techniques as the foundation of the analysis. Each Technique is systematically correlated with: (i) the threat actors (groups & campaigns) known to use it, (ii) the software associated with its execution, (iii) the Tactics under which it is classified, and (iv) the platform on which it operates, with a specific focus on Windows-based systems. Additionally, we incorporate the number of documented mitigations and detections for each technique to provide a clearer assessment of its impact and difficulty. The first step in the proposed approach involves gathering comprehensive data on MITRE ATT&CK techniques, threat groups, campaigns, software, and relationships. This dataset, sourced directly from MITRE, serves as the foundation for our analysis.

### Data collection

The data collection phase focuses on gathering comprehensive cyber threat intelligence from the MITRE TAXII server to support the analysis of tactics, techniques, and procedures (TTPs) within the MITRE ATT&CK framework. This step involves acquiring structured datasets that catalog techniques, adversary groups, attack campaigns, malware/tools, and their interconnections. For example, data on techniques might include their tactical objectives, while relationships link these to specific threat actors. By compiling this information, the process establishes a robust foundation for mapping and analyzing TTPs to enhance cybersecurity threat assessment.

### Data cleaning

The data cleaning phase aims to refine the collected datasets for efficient TTP mapping and analysis. This involves standardizing identifiers for techniques, groups, campaigns, and tools to align with MITRE-defined references, ensuring consistency across the data. This step facilitates seamless integration and querying by eliminating irrelevant metadata and streamlining the datasets. For instance, standardizing technique IDs enables precise linkage to their associated tactics, making the data more accessible for subsequent mapping and improving the accuracy of threat intelligence analysis.

### Data mapping

The data mapping phase seeks to construct a detailed network of relationships between techniques, threat actors, software, and mitigations to inform cybersecurity strategies. Techniques are categorized by their tactical roles, and their usage by threat actors and software is identified through relationship analysis. Additionally, the number of mitigations required to counter each technique is quantified. For example, mapping a technique to a specific malware tool might reveal its deployment by a particular adversary group, enabling targeted defensive measures. This interconnected mapping enhances the understanding of TTPs and supports the development of effective threat mitigation strategies.

The analysis of MITRE ATT&CK data has revealed critical insights into adversary behaviors, particularly in the context of Active Directory (AD) environments. Below, the key findings derived from structured data collection, cleaning, and mapping processes are presented.

A) Most Frequently Used Techniques

**Fig. 4:**
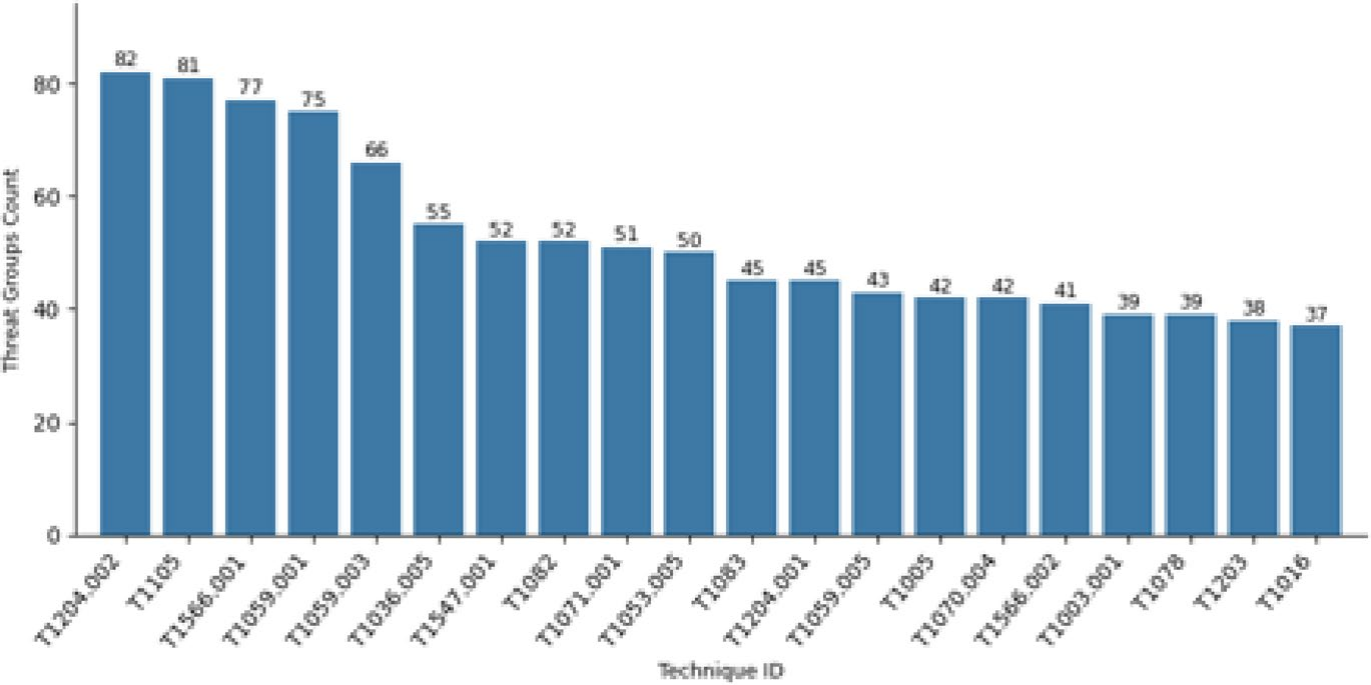
Top 20 Techniques used by Threat Actor Groups.

As techniques represent *how* an adversary achieves a tactical goal by performing an action, our approach is to measure the techniques priority by the number of the threat actors and campaigns that used it. Figure 4 Illustrates top 20 techniques that are used frequently by threat actor groups. The analysis identified that the top 5 techniques in order are : (i) *Malicious File*,* (ii) Ingress Tool Transfer*,* (iii) Spearphishing Attachment*,* (iv) PowerShell*,* (v) Windows Command Shell*.

**Fig. 5:**
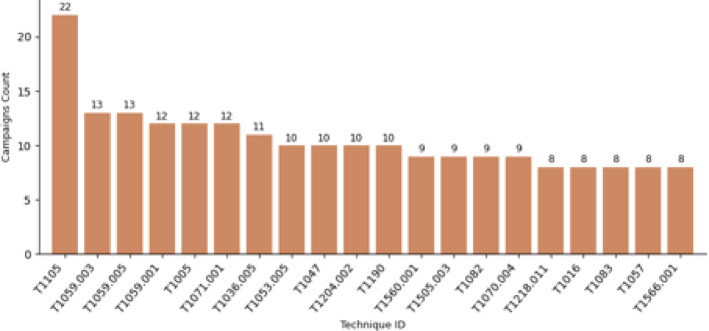
Top 20 Techniques used by Campaigns.

In addition, campaigns have been included as an additional measure of technique adoption. In the subsequent analysis, the counts of threat groups is merged with campaign counts for each technique to achieve a composite measure of usage frequency. The composite measure not only captures the number of threat actors that use a particular technique but also the variety of campaigns through which the technique is used operationally. Figure 5 shows the top 20 techniques used by campaigns. Notably *Ingress Tool Transfer*,* PowerShell*, and *Scheduled task* techniques respectively in the top of use by campaigns.

**Fig. 6:**
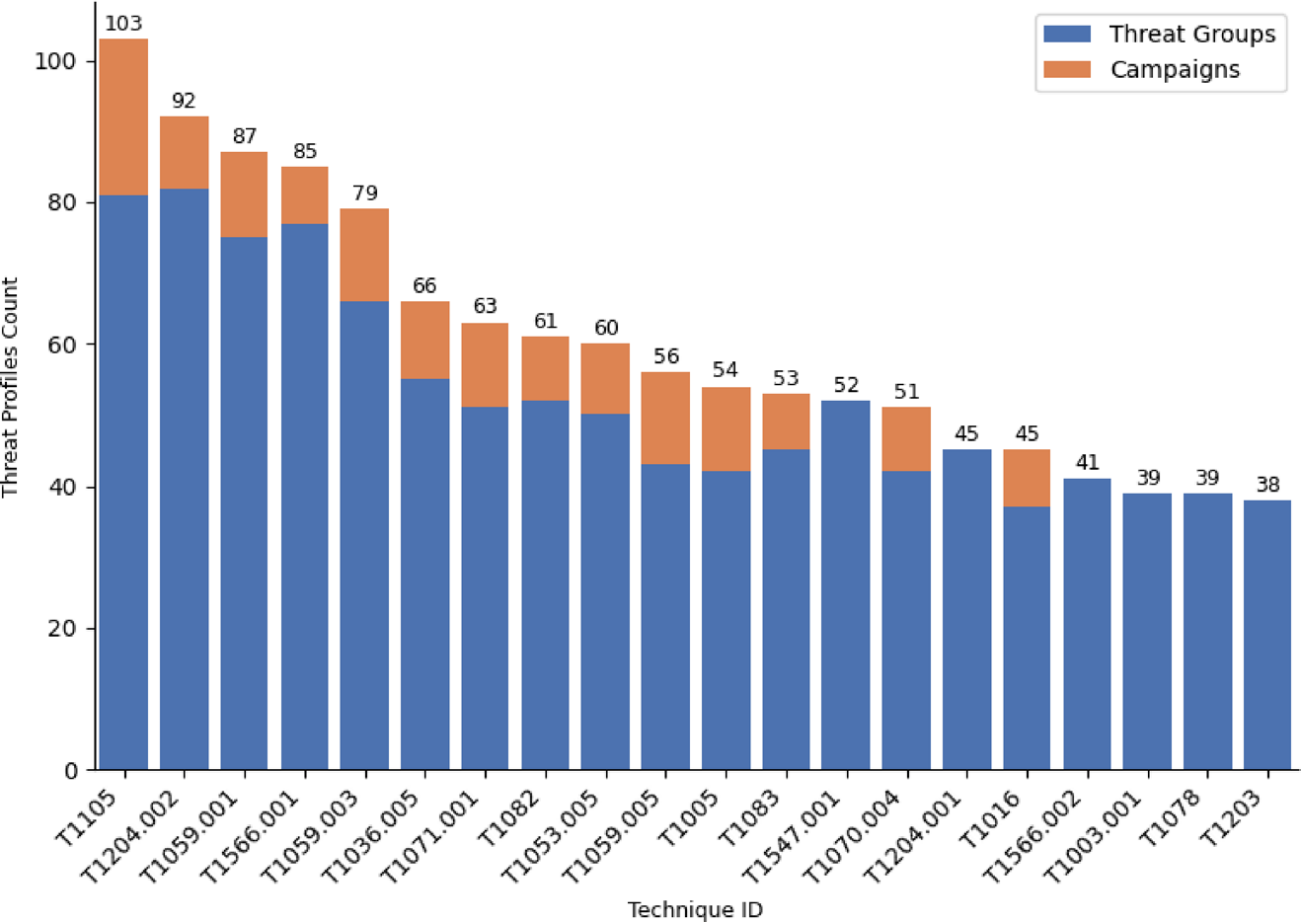
Top 20 Techniques used by Threat Actor Groups and Campaigns.

Figure 6 shows the top 20 techniques ranked by the total frequency of threat groups and campaigns. Notably, the techniques ranked high in the threat group analysis remain prominent even when campaign data are considered. For example, the *Malicious File* and *Ingress Tool Transfer* techniques remain to have the highest overall usage, indicating its prevalence in adversary activities. Further, including campaign numbers adds to the sensitivity of changes in technique prioritization, bringing focus on the operational applicability of some techniques that, while possibly less common within threat groups individually, are highly used within numerous campaigns.

B) Most Prevalent Tactics.

**Fig. 7:**
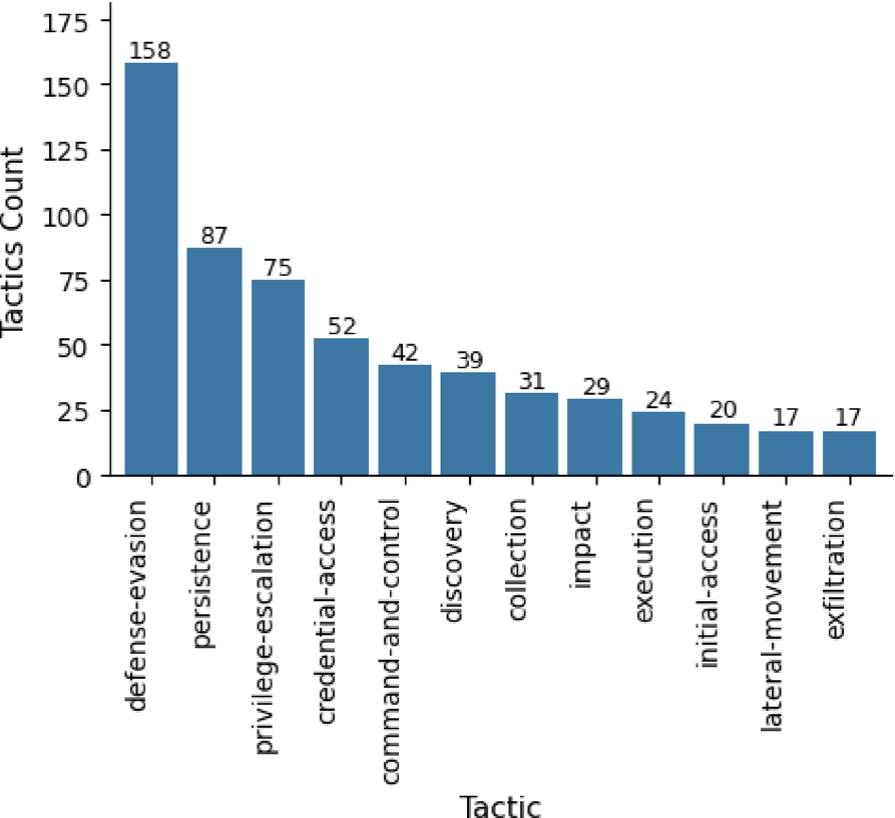
Technique distribution across MITRE ATT&CK tactics.

The analysis of adversary behavior revealed distinct patterns in tactical priorities. As illustrated in Fig. 7, Defense Evasion emerged as the most prevalent tactic, accounting for 26.73% of the observed techniques. This underscores adversaries’ emphasis on bypassing detection mechanisms, through techniques such as *file obfuscation (T1027)* or *clearing logs (T1070).* Following closely, Persistence (14.72%) and Privilege Escalation (12.69%) represented core objectives for maintaining long-term access and expanding control within compromised environments, techniques like *Registry Run Keys (T1547.001)* and *Scheduled Tasks (T1053.005)* were frequently employed to achieve these goals. Credential Access (8.80%) and Command-and-Control (7.11%) emphasized the establishment of reliable communication channels.

C) Most Software used to execute

**Fig. 8:**
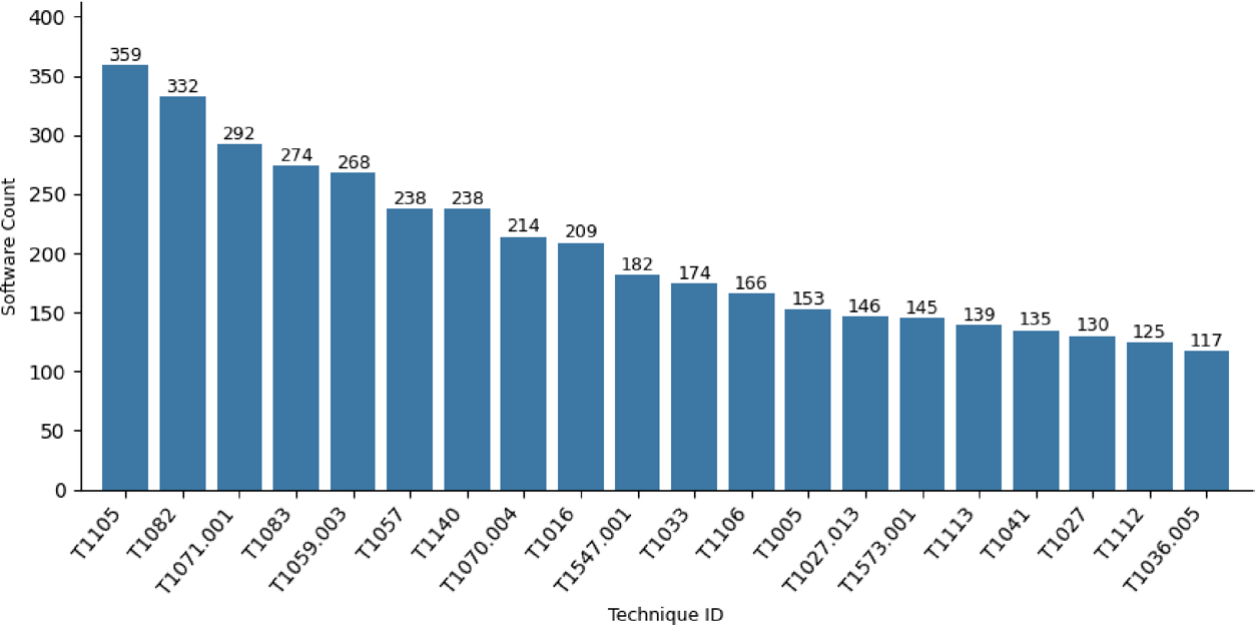
Top 20 Techniques executed by multiple software.

As mentioned, the most challenging portion between the TTPs is the procedure. Moreover, procedures known as the specific implementation the adversary uses for techniques or sub-techniques. A single technique can have multiple software tools associated with it, and each tool can be used in different procedures. Hence, the number of software tools that are associated with the technique reflects the number of possible procedures, and the ability to execute specific technique in multiple ways, and each one has its own IoCs. Therefore, the number of software tools that threat actors use to execute specific techniques is counted. Figure 8 shows the top 20 techniques ranked by the diversity of associated software tools, with *Ingress Tool Transfer (T1105)* leading at 359 tools, followed by *System Information Discovery*,* Web Protocols*,* File and Directory Discovery*, and *Windows Command Shell*.

The dominance of these techniques highlights their operational flexibility. For instance, *T1105 (Ingress Tool Transfer)* supports diverse adversarial workflows from delivering malware via PowerShell scripts to transferring tools over *HTTP/S* or SMB, enabling adversaries to adapt to network defenses. Similarly, *T1082 (System Information Discovery)* is facilitated by tools ranging from native utilities like *systeminfo* to advanced malware like Cobalt Strike, reflecting its role in reconnaissance and privilege escalation.

D) Detection & Mitigation Analysis

**Fig. 9:**
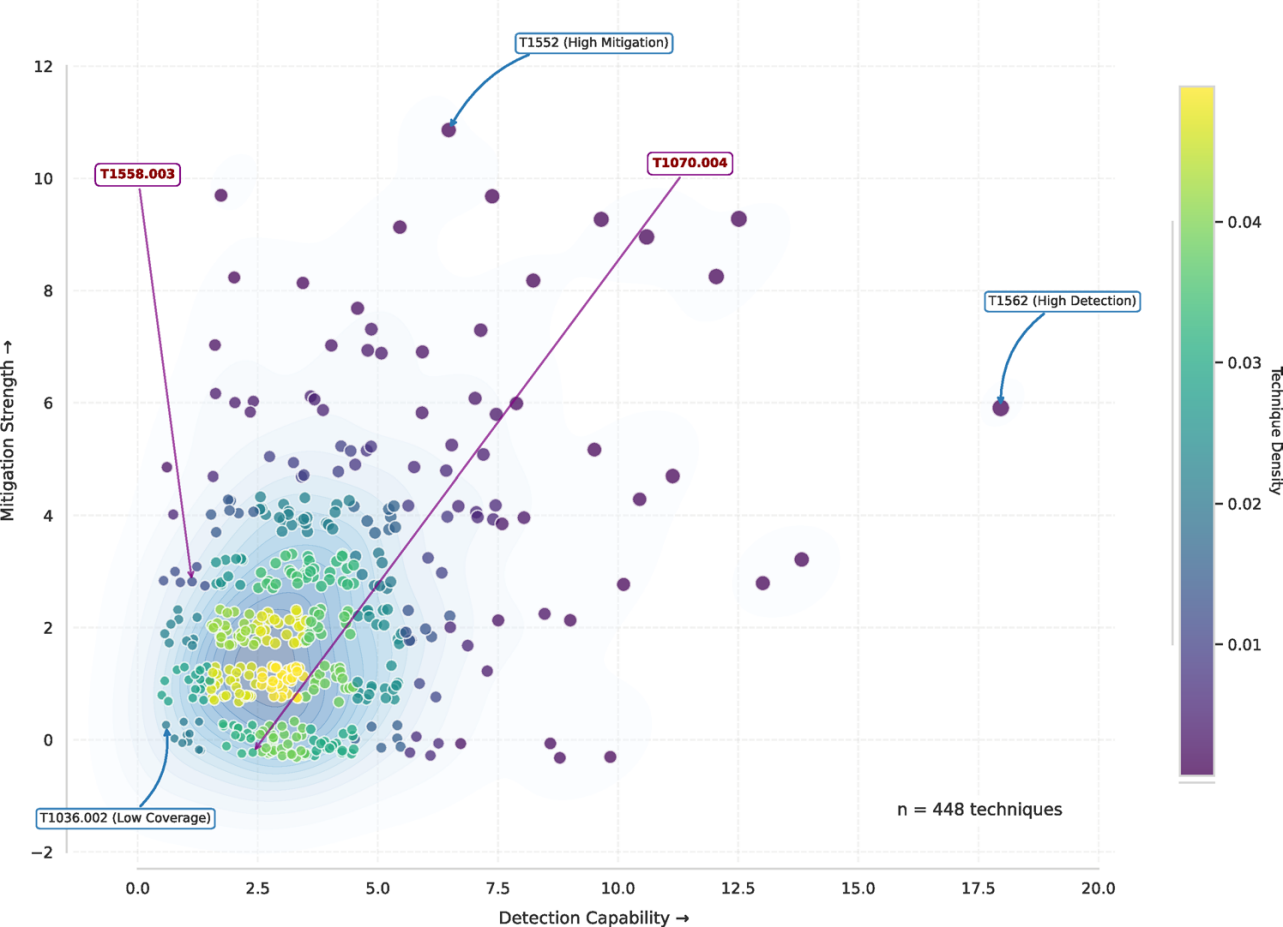
Detection and mitigation coverage across MITRE ATT&CK techniques.

To assess the coverage of the security mechanisms, the number of mitigations and detections associated with each technique is analyzed. Furthermore, to gain insights into the defensive landscape, the total number of associated mitigations and detections is computed, and their distribution is examined, using statistical measures. Finally, the relationship between detection and mitigation counts is evaluated, with Gaussian Kernel Density Estimation (KDE) applied to visualize the density and clustering of techniques in the detection-mitigation space. As shown in Fig. 9. Detection and mitigation coverage across MITRE ATT&CK techniques.,the conducted analysis revealed that some techniques exhibit high detection counts such as *Impair Defenses (T1562)* that got 18 detection capabilities. On the other hand, *Unsecured Credentials (T1552)* which has the most mitigations mechanisms, it has 11 mechanisms to mitigate. Conversely, techniques with low counts for both metrics indicate areas where the current security posture may be particularly vulnerable, such as *Right-to-Left Override* (T1036.002) which has only 1 detection mechanism with no obvious mitigation, making it a persistent evasion threat. Furthermore, as illustrated technique such as *Kerberoasting (T1558.003)* has 3 mitigations but only one detection capabilities, which makes it harder to detect when service tickets are extracted and brute-forced offline. Similarly, *File Deletion (T1070.004)* which is used to cover tracks and facilitate anti-forensics, has no associated mitigations and only two detection mechanisms. This underscores the importance of continuous monitoring and logging to detect and respond to such activities before adversaries erase forensic evidence.

After covering mitigations, detections, and overall defensive coverage, the proposed data-driven prioritization approach is introduced. By integrating multiple data sources, such as detection and mitigation counts, threat actor frequency, and campaign involvement, into a multi-criteria decision-making approach, the proposed approach formally calculates a priority score for each technique. This score reflects both the defensive position and the possible impact of every technique, enabling organizations to focus their efforts on addressing the most critical vulnerabilities.

The following sections outline the mathematical model that forms the foundation of the proposed approach, explain the weighting process based on each organizational risk preferences, and demonstrate how the model applies to real-world data to generate actionable recommendations. The approach not only provides a transparent and repeatable method for TTP prioritization but also adapts to the dynamic nature of the threat landscape, ensuring that adversary emulation exercises remain targeted and effective.

### The proposed prioritization approach using multi-criteria decision-making (MCDM)

Building upon the analysis of the MITRE ATT&CK framework, including technique frequency, tactic prevalence, software diversity, and detection and mitigation coverage, this section introduces the proposed structured, data-driven methodology for prioritizing adversary Tactics, Techniques, and Procedures (TTPs) within Active Directory (AD) environments. Traditional adversary emulation approaches often lack a systematic mechanism to determine which techniques should be prioritized for evaluation, leaving organizations with an overwhelming number of possible attack scenarios. To address this challenge, a Multi-Criteria Decision-Making (MCDM) model is proposed to integrate multiple critical factors into adversary emulation.

Our approach leverages three primary dimensions: (i) *Active Directory Impact Score (*$\:{C}_{1})$ which evaluates the potential consequences of a technique based on and difficulty of mitigation; (ii) *Threat Score*$\:\left({C}_{2}\right)$, which incorporates real-world threat intelligence, exploitability, and observed prevalence of a technique in attacks; and (iii) *Security Control Gap*$\:\left({C}_{\text{3}}\right)$, which assesses the effectiveness of existing defensive mechanisms in detecting and mitigating the technique. By systematically aggregating these dimensions, we compute a Final *Priority Score*$\:\:\left({P}_{S}\right)$, which serves as a quantifiable measure to rank techniques based on their criticality.

This prioritization approach ensures that adversary emulation efforts are not only guided by theoretical attack frameworks but are also informed by empirical security intelligence and an organization’s defensive posture. By formalizing the prioritization process through MCDM, our approach provides a transparent, repeatable, and context-aware methodology for selecting high-impact techniques, optimizing security testing, and enhancing the resilience of Active Directory infrastructures.

## Criteria identification and selection

In the first phase, we identify and select the key criteria that will serve as the foundation for our prioritization model. Our approach is based on the observation that not all techniques pose the same level of risk or demand the same defensive resources in an Active Directory (AD) context. Accordingly, we define three primary dimensions:

1 $$\:{C}_{1}=\frac{1}{{Mitigation\:Count}+1}\hspace{1em}$$

1) Active Directory Impact $\:\left({C}_{1}\right)$.

This criterion assesses the potential severity or consequence if a technique is successfully executed within the AD environment. We operate this by considering the difficulty of mitigating the technique. Techniques with fewer documented MITRE mitigations are considered harder to prevent and thus potentially more impactful. A higher score indicates higher potential impact, calculated as follows:

2) Threat Score $$\:\left({C}_{2}\right)$$.

This criterion reflects how commonly a technique is observed in real-world attacks, indicating its relevance based on operational threat intelligence. We use a composite measure based on the number of threat groups, campaigns, and software (malware/tools) associated with the technique in the ATT&CK knowledge base. A higher score indicates greater threat relevance, and calculated as follows:

2 $$\:{C}_{2}={ln}\left({threat\:groups\:count}+1\right)+{ln}\left({campaign\:counts}+1\right)+{ln}\left({software\:count}+1\right)\hspace{1em}$$

3) Security Control Gap $\:\left({C}_{3}\right)$.

This criterion assesses the difficulty in detecting the execution of a technique, representing gaps in security controls or visibility. Techniques with fewer documented MITRE detection methods are considered harder to detect, indicating a larger control gap. A higher score indicates a larger gap. The formula is:

3 $$\:{C}_{3}=\frac{1}{{Detection\:Count}+1}\hspace{1em}$$

## Entropy weight method for resources weighting

In our approach of weighting, we use *Entropy Weight Method (EWM)* to avoid the interference of human factors on the weight of indicators, thus enhancing the objectivity of the comprehensive evaluation results^37^. The Entropy Weight Method (EWM) is employed to determine the objective weights of the criteria based on the inherent information present in the decision matrix. It assumes that criteria exhibiting greater variability across alternatives provide more information and should thus receive higher weights. The steps are as follows:

**Step 1: Normalize the Decision Matrix.**

Let $\:\:X\:=\:{\left[{x}_{\left\{ij\right\}}\right]}_{\left(\left\{m\:\times\:n\right\}\right)}$be the decision matrix, where $\:{x}_{ij}$ is the score of the $\:{i}_{th}$ technique (alternative) on the $\:{j}_{th}$ criterion. Normalize the matrix to obtain $\:{p}_{ij}$ using the following formula for benefit criteria:

4 $$\:{p}_{ij}=\frac{{x}_{ij}}{{\sum\:}_{j=1}^{n}{x}_{ij}}\hspace{1em}$$

**Step 2: Calculate the Entropy** $\:{\varvec{E}}_{\varvec{i}}$ **for each criterion.**

The entropy measures the degree of uncertainty or divergence in the data for a criterion. It is calculated as:

5 $$\:{E}_{i}=-\frac{{\sum\:}_{j=1}^{n}{p}_{ij}\cdot\:{ln}{p}_{ij}}{{ln}n}\hspace{1em}$$

$$(\text{where } k = \frac{1}{\ln(m)} \text{ is a constant ensuring } 0 \le E_j \le 1. \text{ If } p_{ij} = 0, \text{ then } p_{ij} \ln(p_{ij}) \text{ is taken as } 0.)$$

A higher entropy value indicates greater variability in the data, meaning the criterion provides more information and should be assigned a higher weight. The entropy value $\:{E}_{i}\:$ranges from 0 to 1. When $\:{E}_{i}$ has a larger value, index $\:i\:$ exhibits a higher degree of differentiation, providing more information.

**Step 3: Calculate the Degree of Divergence** $\:{\varvec{d}}_{\varvec{j}}$ **.**

The divergence $\:{\varvec{d}}_{\varvec{j}}$ represents the amount of useful information provided by criterion

6 $$\:{d}_{j}=\:1\:-\:{E}_{j\hspace{1em}}$$

$$(\text{A higher } d_j \text{ indicates greater variability and thus more information content for criterion } j.)$$

**Step 4: Calculate the Objective Weight** $\:{w}_{\varvec{j}}$ **for each criterion** $\:j$.

The weights are derived by normalizing the divergence values:

7 $$\:{w}_{i}=\frac{1-{E}_{i}}{{\sum\:}_{i=1}^{m}\left(1-{E}_{i}\right)}\hspace{1em}$$

## Weighted sum method for aggregation and ranking

The Weighted Sum Method (WSM) is a simple and widely used MCDM technique for aggregating scores. It calculates a final score $\:P{S}_{i}$ for each technique $\:i$ by multiplying its normalized score on each criterion by the criterion’s weight and summing the results.

**Step 1: Use the normalized matrix**.

Alternatively, if a different normalization (like vector normalization or min-max) is preferred for WSM, apply it here. For consistency, we can use the same normalization as EWM:

8 $$\:{p}_{ij}=\frac{{x}_{ij}}{{\sum\:}_{j=1}^{n}{x}_{ij}}\hspace{1em}$$

Step 2: Calculate the final score $\:P{S}_{i}$ for each technique.

9 $$\:P{S}_{i}={\sum\:}_{j=1}^{3}{w}_{j}\cdot\:{p}_{ij}\hspace{1em}$$

Where $\:{w}_{j}$ are the entropy weights calculated in EWM Step 4.

While the prioritization scores $\:P{S}_{i}$ derived from the Weighted Sum Method (Eq. 8) reflect the true aggregated utility of each technique under the entropy-based.

weighting scheme, they may not lie within a bounded range such as [0,1]. For improved interpretability and comparative visualization, a min–max normalization step can be applied to rescale the priority scores:

$$\:P{S}_{i}^{\text{norm}}=\frac{P{S}_{i}-\text{min}\left(PS\right)}{\text{max}\left(PS\right)-\text{min}\left(PS\right)}$$

This transformation does not alter the relative ranking of techniques and is used solely for presentation purposes. It ensures that the highest-priority technique is scaled to 1 and the lowest to 0, without affecting the underlying entropy-based evaluation process.

**Step 3: Rank the techniques.**

Rank the techniques in descending order based on their final scores $\:{S}_{i}$​. The technique with the highest score is considered the highest priority.

- A high $\:P{S}_{i}$ indicates a technique that is impactful, widely used, and less detectable, making.

it a high-priority target for defensive measures.

- A low $\:P{S}_{i}$ suggests a technique that is less critical and easier to mitigate.

**Fig. 10:**
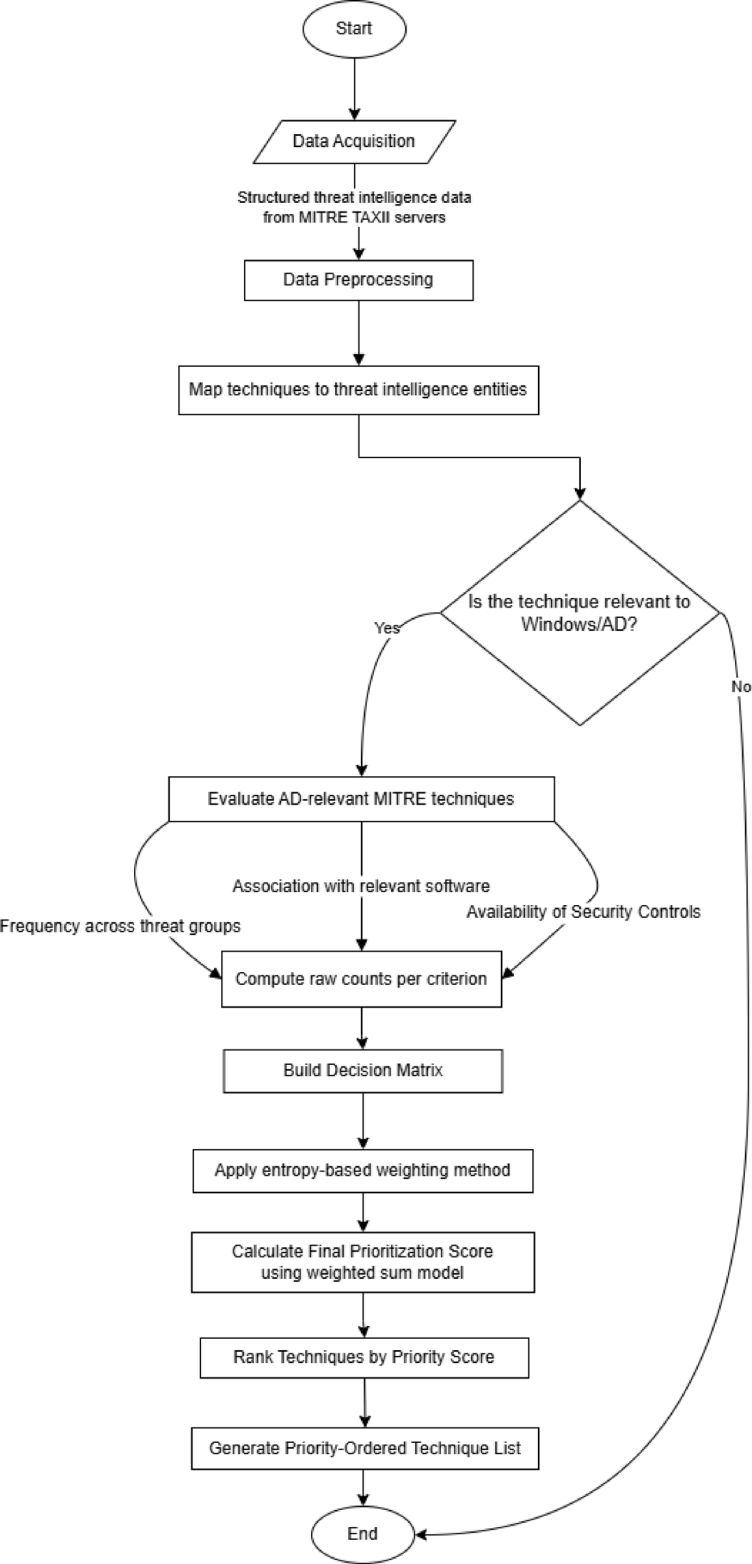
Workflow of the Proposed Data Acquisition and Technique Prioritization Pipeline.

To provide a clear overview of the structured methodology adopted in this research, a flowchart is presented in Fig. 10. This workflow visualizes the end-to-end process used to collect, clean, map, and prioritize adversary Tactics, Techniques, and Procedures (TTPs) within the MITRE ATT&CK framework, with a focus on Windows-based environments and Active Directory. The process begins with the acquisition of raw cyber threat intelligence from the MITRE TAXII server, followed by rigorous data cleaning to standardize and normalize entities such as technique identifiers, threat groups, campaigns, and software. Subsequently, the cleaned data is mapped to establish meaningful relationships between techniques, associated threat actors, software tools, and mitigations. This mapping phase enables a deeper contextual understanding of TTP deployment across real-world adversary operations. Finally, a prioritization step integrates platform specificity, threat relevance to Active Directory, and the presence of mitigations and detections to generate a refined, actionable dataset. The flowchart encapsulates this systematic approach, serving as a visual guide for the methodology employed in this study.

To elucidate the methodology prior to its application in Sect. 4, we present a concise, illustrative example involving three synthetic techniques. This demonstration comprises three stages: (i) computation of raw criterion scores based on control gap, threat prevalence, and detection evasion; (ii) normalization of the decision matrix using the same proportional (vector) normalization applied in the Entropy Weight Method (EWM); and (iii) aggregation of the normalized scores via the Weighted Sum Method (WSM), using entropy-derived weights to compute a final prioritization score for each technique.

### Example 1

Let T = {A, B,C) be a set of techniques characterized by their mitigation count, detection count, and associated threat intelligence. The raw attribute counts are presented in Table 4 :

**Table 4:** Hypothetical Raw counts.

Technique ID	Mitigation Count	Detection Count	Groups Count	Campaigns Count	Software Count
A	1	3	10	4	20
B	4	1	5	2	10
C	0	2	2	1	5

**Step 1. Compute raw criterion scores.**

1- **Impact** $\:{C}_{1}$ : inversely proportional to the number of mitigations

$$\:{C}_{1}=\frac{1}{{Mitigation\:Count\:+\:1}}\hspace{1em}$$

2- **Prevalence** $\:{C}_{2}$ : the sum of associated threat groups, campaigns, and software

$$\:{C}_{2}\left(t\right)={\text{Groups}}_{t}+{\text{Campaigns}}_{t}+{\text{Software}}_{t}$$

3- **Security Control Gap** $\:{C}_{3}$ : inversely proportional to the number of detections

$$\:{C}_{3}=\frac{1}{{Detection\:Count}+1}\hspace{1em}$$

The resulting criterion scores are shown in Table 5.

**Table 5:** Raw criterion scores.

Technique ID	$\:{\varvec{C}}_{1}\varvec{}=1/\left(\varvec{m}+1\right)$	$\:{\varvec{C}}_{2}\varvec{}=\varvec{g}+\varvec{c}+\varvec{s}$	$\:{\varvec{C}}_{3}\varvec{}=1/\left(\varvec{d}+1\right)$
$\:{T}_{A}$	0.50	7.052	0.25
$\:{T}_{\text{B}}$	0.20	5.289	0.50
$\:{T}_{\text{C}}$	1.00	3.584	0.33

**Step 2. Normalize the Decision Matrix**.

To enable entropy-based analysis, the matrix is column-normalized as follows:

$$\:{r}_{ij}=\frac{{x}_{ij}}{{\sum\:}_{i=1}^{m}{x}_{ij}}$$

The normalized scores are presented in Table 6.

**Table 6:** Normalized decision matrix.

Technique ID	$\:{\varvec{r}}_{\varvec{i}\varvec{j}}\left({\varvec{C}}_{1}\right)$	$\:{\varvec{r}}_{\varvec{i}\varvec{j}}\left({\varvec{C}}_{2}\right)$	$\:{\varvec{r}}_{\varvec{i}\varvec{j}}\left({\varvec{C}}_{3}\right)$
$\:{T}_{A}$	0.294	0.443	0.231
$\:{T}_{\text{B}}$	0.118	0.332	0.462
$\:{T}_{\text{C}}$	0.588	0.225	0.307

**Step 3. Compute Criterion Entropy**.

Entropy is computed using the standard formula:

$$\:{e}_{j}=-k{\sum\:}_{i=1}^{m}{r}_{ij}\text{ln}{r}_{ij}\:\:\:\:\:\:where\:k=\frac{1}{\text{ln}\left(m\right)}$$

The results are:

•$$\:{e}_{1}\approx\:0.870$$

•$$\:{e}_{2}\approx\:0.877$$

•$$\:{e}_{3}\approx\:0.995$$

**Step 4. Compute Diversification and Weights**.

The degree of diversification for each criterion is given by:

$$\:{d}_{j}=1-{e}_{j}$$

The resulting diversification values:

•$$\:{d}_{1}\approx\:0.130$$

•$$\:{d}_{2}\approx\:0.123$$

•$$\:{d}_{3}\approx\:0.005$$

Weights are then derived through normalization:

$$\:{w}_{j}=\frac{{d}_{j}}{{\sum\:}_{j}{d}_{j}}$$

This yields:

•$$\:{w}_{1}\approx\:0.51$$

•$$\:{w}_{2}\approx\:0.48$$

•$$\:{w}_{3}\approx\:0.02$$

**Step 5. Vector Normalization for WSM**.

To maintain consistency, the same normalized matrix from Step 2 (Table 6) is reused for scoring via the Weighted Sum Method (WSM).

**Step 6. Final Score Using WSM**.

Each technique’s final priority score is computed by aggregating the normalized scores with their respective entropy weights:

$$\:P{S}_{i}={\sum\:}_{j=1}^{3}{w}_{j}\cdot\:{p}_{ij}$$

The calculations and final scores are summarized in Table 7.

**Table 7:** Final priority scores.

Technique ID	$\:\varvec{P}{\varvec{S}}_{\varvec{i}}$
$\:{T}_{A}$	0.364
$\:{T}_{\text{B}}$	0.226
$\:{T}_{\text{C}}$	0.409

**Step 7. Ranking Techniques**.

Rank the techniques by descending $\:{S}_{i}$ :

• $$\:{T}_{C}$$ : 0.409 ◊ Highest Priority.

• $$\:{T}_{\text{A}}$$ : 0.364 ◊ Medium Priority.

• $$\:{T}_{\text{B}}$$ : 0.226 ◊ Lowest Priority.

## Case study : APT3 emulation

APT3, also known as Gothic Panda or UPS, is a well-documented advanced persistent threat (APT) group believed to have ties to China^38^. This group is known for targeting a variety of sectors, including technology, government, and defense industries. APT3 employs a range of sophisticated tactics, techniques, and procedures (TTPs) to infiltrate and maintain persistence within target networks. The group’s activities have been extensively documented in open-source references, including the MITRE ATT&CK framework, which provides a comprehensive Adversary Emulation Plan for APT3^38^. Nevertheless, in^38^, the extracted TTPs were outdated, the group continued its adversarial attacks, so for the experiment, the techniques were derived from the up-to-date dataset that has been extracted previously through the process of MITRE ATT&CK analysis. These techniques were then mapped to the MITRE ATT&CK framework and aligned with the necessary criteria for subsequent calculations, as presented in Table 8. The extracted techniques which used by APT3 threat actor group indicates that they used 45 techniques throughout their historical adversarial attacks.

Data for the three prioritization criteria (defined in Sect. 3.3) were operationalized using these counts:

1. Potential Impact ($\:{C}_{1}$): Calculated as $\:{C}_{1}=\frac{1}{\text{Mitigation Count + }\text{1}}$. Techniques with fewer mitigations receive a higher score.

2. Threat Relevance ($\:{C}_{2}$): Calculated using a log-transformed sum:

$$ C_2 = \ln(\text{threat groups count} + 1) + \ln(\text{campaign counts} + 1) + \ln(\text{software count} + 1) $$

Log transformation normalizes the distribution and reduces skewness from potentially large counts.

3. Control Efficacy Gap ($\:{C}_{3}$): Calculated as $\:{C}_{3}=\frac{1}{\text{Detection Count}+1}\:$. Techniques with fewer detection methods receive a higher score, indicating a larger gap.

**Table 8:** Mapping APT3 TTPs to MITRE and to the approach criteria.

Technique ID	Mitigation Count	Detection Count	Groups Count	Campaigns Count	Software Count
T1056.001	0	3	25	3	116
T1053.005	4	7	50	10	101
T1560.001	1	3	34	9	25
T1033	0	9	36	6	174
T1218.011	1	4	24	8	66
T1546.008	3	5	6	0	1
T1016	0	4	37	8	209
T1049	0	3	30	3	61
T1083	0	3	45	8	274
T1136.001	2	3	14	0	15
T1555.003	5	4	22	1	56
T1090.002	1	3	11	0	10
T1003.001	7	7	39	6	24
T1547.001	0	5	52	2	182
T1027.005	0	1	6	2	9
T1104	1	2	4	0	9
T1552.001	4	3	14	0	17
T1059.001	5	5	75	12	108
T1041	2	5	21	6	135
T1057	0	3	36	8	238
T1087.001	1	5	15	1	41
T1543.003	5	10	22	4	100
T1566.002	5	3	41	7	26
T1069	0	5	5	1	6
T1110.002	2	2	3	1	1
T1074.001	0	4	24	6	77
T1021.002	4	6	23	5	24
T1082	0	3	52	9	332
T1098.007	0	1	7	0	4
T1005	1	5	42	12	153
T1564.003	2	4	14	0	30
T1078.002	5	3	16	6	5
T1059.003	1	2	66	13	268
T1036.010	2	1	3	1	2
T1027	4	10	18	3	130
T1095	3	2	9	6	74
T1203	3	4	38	2	13
T1204.001	3	3	45	7	27
T1027.002	1	1	21	6	66
T1070.004	0	2	42	9	214
T1021.001	8	5	31	6	16
T1574.002	2	4	24	2	37
T1105	1	5	81	22	359
T1018	0	4	35	7	48

**Table 9:** APT3 techniques sorted by priority.

Technique ID	Name	Priority score
T1027.005	Indicator Removal from Tools	1
T1098.007	Additional Local or Domain Groups	0.97
T1070.004	File Deletion	0.96
T1082	System Information Discovery	0.90
T1083	File and Directory Discovery	0.89
T1057	Process Discovery	0.88
T1056.001	Keylogging	0.86
T1049	System Network Connections Discovery	0.85
T1016	System Network Configuration Discovery	0.84
T1018	Remote System Discovery	0.82
T1074.001	Local Data Staging	0.82
T1547.001	Registry Run Keys/Startup Folder	0.80
T1033	System Owner/User Discovery	0.75
T1069	Permission Groups Discovery	0.71
T1027.002	Software Packing	0.63
T1059.003	Windows Command Shell	0.54
T1105	Ingress Tool Transfer	0.42
T1560.001	Archive via Utility	0.42
T1104	Multi-Stage Channels	0.41
T1005	Data from Local System	0.39
T1036.010	Masquerade Account Name	0.39
T1218.011	Rundll32	0.39
T1090.002	External Proxy	0.36
T1087.001	Local Account	0.32
T1095	Non-Application Layer Protocol	0.27

Finally, after applying the MCDM methodology, the calculated scores, derived from Table 8, from the input decision matrix $\:{X}_{45\times\:3}$ for the proposed methodology described in Sect. 3.3. As shown in Table 9, how the approach applied on the APT3 techniques and successfully sorted the techniques.

### Validation and observations

To validate the correctness of the calculations, a step-by-step verification of the entropy-based weighting and score computation was performed. The entropy weights for the three primary criteria were computed as follows: C₁ (Active Directory Impact) received a weight of 0.592, C₂ (Threat Score) received 0.163, and C₃ (Security Control Gap) received 0.245. These weights ensured that techniques with low mitigation coverage, high threat actor adoption, and significant security control deficiencies were appropriately emphasized in the prioritization process.

The Threat Score (C₂) was derived by applying entropy-based weights to its three sub-criteria: threat actor adoption, campaign prevalence, and software diversity. The logarithmic transformation of raw values prevented extreme skewness, ensuring a balanced representation of threat prevalence. The Security Control Gap Score (C₃) was adjusted to reflect both theoretical best practices from MITRE ATT&CK recommendations and the actual implemented security controls in an environment. This adjustment ensured that techniques with highly recommended controls, but low actual implementation were prioritized, guiding organizations to address their most significant security gaps.

A systematic comparison was conducted between the highest-ranked techniques produced by the proposed EWM–WSM prioritization framework and the known behavioral patterns of APT3, as documented in the MITRE ATT&CK database. The analysis revealed a strong convergence between the model’s output and empirically observed adversarial tactics, particularly within the domains of discovery, execution, and anti-forensics. Notably, many of the top-scoring techniques correspond to early-stage reconnaissance, lateral movement, and covering tracks activities that are critical for post-compromise progression in Active Directory (AD) environments.

This prioritization highlights a salient operational insight: defenders should focus on monitoring discovery and enumeration commands, particularly when executed by non-administrative or anomalous user accounts. For instance, the execution of interface or network discovery tools by HR personnel may serve as an indicator of compromise, given the incongruence with expected user behavior.

**Top-ranked techniques aligned with APT3**.

• T1027.005 – Indicator Removal from Tools, with the highest priority reflects APT3’s emphasis on anti-forensics and stealth. This technique, which facilitates the deletion of logs or artifacts, underscores the importance of evasion in post-access operations.

• T1098.007 – Additional Local or Domain Groups, is frequently used by adversaries Often leveraged for enumeration and privilege escalation in domain environments, particularly valuable for adversaries targeting group membership for lateral movement.

• T1070.004 – File Deletion, is a classic anti-forensic measure. Its high rank is attributed to a lack of effective mitigations and broad support across malware families, making it an efficient method for attackers to conceal activity.

Moreover, techniques such as T1082 – System Information Discovery, File and Directory Discovery and T1057 – Process Discovery were all highly ranked due to their foundational role in APT3’s host profiling and reconnaissance phase. These techniques are widely used to collect system-level insights prior to privilege escalation or lateral movement, and their elevated scores emphasize their continued relevance in emulation planning.

While the top-priority techniques demonstrated strong alignment with APT3’s historical attack patterns, the analysis also revealed certain lower-ranked techniques that have been observed in previous APT3 attacks. For example:

• T1095 – Non-Application Layer Protocol ranked notably low, primarily due to its relatively narrow threat applicability and increased visibility. Its usage often involves non-standard communication patterns that are now more readily flagged by mature network security monitoring systems.

• T1087.001 – Local Account was also deprioritized. While credential access remains a critical threat vector, this specific sub-technique is often associated with noisy or privilege-requiring actions, making it less practical in stealth-centric post-compromise phases. Additionally, these actions typically trigger well-defined logs or authentication alerts within modern EDR/SIEM platforms.

The primary implication of this research is the provision of a structured, data-driven, and transparent methodology for prioritizing TTPs in adversary emulation planning, specifically tailored for AD environments. By applying the EWM-WSM framework, security teams can move beyond ad-hoc selection, generic scenarios, or simple frequency counts, focusing their limited resources on emulating techniques that are demonstrably more critical based on a balanced assessment of potential impact, threat relevance, and control gaps.

This prioritization directly addresses the problem of choosing critical techniques. Instead of attempting superficial coverage of a wide range of TTPs, the framework guides teams to concentrate on the top-ranked techniques. Emulating these high-priority TTPs allows for a deeper assessment of defenses against the most pertinent threats, leading to more effective identification of vulnerabilities and targeted improvements in detection and response capabilities. The use of objective weighting (EWM) removes the subjectivity inherent in manually assigning weights based on intuition, lending greater credibility and repeatability to the prioritization process. The framework’s reliance on quantifiable data derived from MITRE ATT&CK ensures that prioritization is grounded in widely accepted operational threat intelligence.

### Adaptability of prioritization

To demonstrate the theoretical robustness of the proposed Multi-Criteria Decision-Making (MCDM) framework, it is essential to analyze how prioritization outcomes respond to variations in threat actor profiles and shifts in organizational risk criteria. This discussion highlights the approach’s conceptual flexibility, extending beyond applied scenarios to its foundational design.

Technique prioritization is inherently dynamic, shaped by the operational characteristics of the adversary in question. From a theoretical standpoint, the approach’s adaptability to different APT groups (e.g., APT3 vs. APT29) derives from its integration of adversary-specific Operational Threat Intelligence (OTI) into the “Threat Score” criterion. Since each group exhibits a distinct distribution of TTPs, changes in the underlying OTI dataset yield corresponding shifts in threat scores, which propagate through the weighted sum aggregation to re-rank techniques. This data-driven recalibration ensures that prioritization reflects the most statistically significant and operationally relevant TTPs for the adversary under consideration, underscoring the model’s capacity for dynamic alignment based on evolving empirical evidence.

Similarly, the approach is designed to accommodate internal organizational priorities through its modular, weight-adjustable structure. The core criteria, Active Directory Impact, Threat Score, and Security Control Gap, capture distinct dimensions of risk, with initial weights derived via the Entropy Weight Method (EWM). However, these weights can be theoretically adjusted to reflect evolving strategic emphasis. For example:

• Shifting Risk Priorities: An increased concern for operational resilience may warrant greater weight on “Active Directory Impact” or the inclusion of a new criterion, such as “Operational Disruption Potential.”

• Expanding Risk Dimensions: The model permits the seamless integration of additional criteria, such as “Regulatory Compliance Risk” or “Reputational Impact”, provided these can be appropriately quantified and normalized.

This extensibility reinforces the approach’s theoretical soundness: the weighted sum formulation readily accommodates modifications in both input data and criterion structure, allowing the framework to evolve with changing adversarial contexts and institutional risk postures.

In essence, the adaptability of the approach is not incidental, but a direct outcome of its theoretical architecture, a mathematically grounded, decision-theoretic model capable of systematic reconfiguration in response to both external threat dynamics and internal policy shifts. This foundational flexibility ensures its sustained applicability across diverse cybersecurity use cases and evolving operational environments.

## Conclusion and future work

Despite MITRE ATT&CK’s comprehensive coverage of adversarial behaviors, current emulation strategies often rely on static playbooks or subjective choices. These methods overlook real-time threat intelligence, control gaps, and evolving attack trends, leading to misaligned or outdated TTP selection. This research addresses these gaps with a structured, data-driven framework that integrates Cyber Threat Intelligence (CTI) and Multi-Criteria Decision-Making (MCDM). By combining Active Directory Impact, Threat Score, and Security Control Gaps using entropy-based weighting, the model prioritizes TTPs that are both high-risk and under-defended.

The model synthesizes three core criteria, Active Directory Impact, Threat Prevalence, and Security Control Gaps, and applies entropy-based weighting and the Weighted Sum Method (WSM) to generate objective, context-aware rankings of ATT&CK techniques. The methodology is both flexible and extensible, offering a rigorous yet practical decision-support tool for adversary emulation planning.

Key contributions include:

• Context-aware mapping techniques to adversary behavior, mitigations, and visibility gaps.

• Quantitative ranking of TTPs to support focused, resource-efficient emulation exercises.

• Demonstrated effectiveness in highlighting critical AD-related TTPs using real-world data.

Moreover, this research extends beyond its immediate practical utility to offer a substantive theoretical contribution to the academic discipline of cybersecurity. By systematically integrating Multi-Criteria Decision-Making (MCDM) methodologies with the MITRE ATT&CK framework, the study introduces a novel analytical paradigm for interpreting and prioritizing adversarial behaviors. This approach transcends conventional descriptive or empirical treatments by establishing a mathematically rigorous foundation for threat modeling and cyber risk assessment. Specifically, it demonstrates how complex, multi-dimensional threat phenomena can be formally deconstructed, weighted, and evaluated within a structured decision-theoretic framework. In doing so, the work advances the theoretical underpinnings of cybersecurity analytics, enriches scholarly discourse on decision-making under uncertainty, and lays the groundwork for future methodological developments at the intersection of operational research and cyber defense.

One area for enhancement in the current approach is its reliance on static datasets, which may not fully capture the evolving threat landscape or newly emerging techniques. To address this, future work will focus on integrating real-time cyber threat intelligence through APIs (e.g., STIX/TAXII-compatible feeds), enabling the system to maintain relevance through continuous updates. Additionally, adopting a rolling-window approach that incorporates data from the most recent 12 months will improve temporal relevance and strengthen the system’s adaptability to rapidly changing adversarial behaviors.

Further directions include:

1. **Refine Criteria**: Add factors like mitigation effectiveness or asset criticality using hybrid MCDM.

2. **Enable Dynamic Updates**: Integrate real-time CTI and posture changes.

3. **Incorporate Attack Paths**: Prioritize based on TTP chains, not just individual techniques.

4. **Automate Tooling**: Develop open-source tools for data ingestion, scoring, and emulation via Caldera or Atomic Red Team.

## Supplementary Information

Below is the link to the electronic supplementary material.

Supplementary Material 1

Supplementary Material 2

Supplementary Material 3

Supplementary Material 4

Supplementary Material 5

Supplementary Material 6

Supplementary Material 7

Supplementary Material 8

Supplementary Material 9

Supplementary Material 10

Supplementary Material 11

## Data Availability

Our data and codes are provided at https://github.com/0xmahmoudJo0/MITRE-Analysis-Prioritization.
